# Arf6 controls beta-amyloid production by regulating macropinocytosis of the Amyloid Precursor Protein to lysosomes

**DOI:** 10.1186/s13041-015-0129-7

**Published:** 2015-07-14

**Authors:** Weihao Tang, Joshua H.K. Tam, Claudia Seah, Justin Chiu, Andrea Tyrer, Sean P. Cregan, Susan O. Meakin, Stephen H. Pasternak

**Affiliations:** J. Allyn Taylor Centre for Cell Biology, Molecular Medicine Research Group, Robarts Research Institute, 1151 Richmond St, London, ON N6A 5B8 Canada; Department of Clinical Neurological Sciences, Schulich School of Medicine, the University of Western Ontario, London, ON N6A 5B7 Canada; Department of Physiology and Pharmacology, Schulich School of Medicine, the University of Western Ontario, London, ON N6A 5B7 Canada; Department of Biochemistry, Schulich School of Medicine, the University of Western Ontario, London, ON N6A 5B7 Canada

## Abstract

Alzheimer’s disease (AD) is characterized by the deposition of Beta-Amyloid (Aβ) peptides in the brain. Aβ peptides are generated by cleavage of the Amyloid Precursor Protein (APP) by the β − and γ − secretase enzymes. Although this process is tightly linked to the internalization of cell surface APP, the compartments responsible are not well defined. We have found that APP can be rapidly internalized from the cell surface to lysosomes, bypassing early and late endosomes. Here we show by confocal microscopy and electron microscopy that this pathway is mediated by macropinocytosis. APP internalization is enhanced by antibody binding/crosslinking of APP suggesting that APP may function as a receptor. Furthermore, a dominant negative mutant of Arf6 blocks direct transport of APP to lysosomes, but does not affect classical endocytosis to endosomes. Arf6 expression increases through the hippocampus with the development of Alzheimer’s disease, being expressed mostly in the CA1 and CA2 regions in normal individuals but spreading through the CA3 and CA4 regions in individuals with pathologically diagnosed AD. Disruption of lysosomal transport of APP reduces both Aβ40 and Aβ42 production by more than 30 %. Our findings suggest that the lysosome is an important site for Aβ production and that altering APP trafficking represents a viable strategy to reduce Aβ production.

**Electronic supplementary material:**

The online version of this article (doi:10.1186/s13041-015-0129-7) contains supplementary material, which is available to authorized users.

## Introduction

Alzheimer’s disease (AD) is a progressive neurodegenerative disease that is characterized by the deposition of beta-amyloid (Aβ) peptides in plaques in the brain. Aβ is produced by the sequential cleavage of the Amyloid Precursor Protein (APP). The first cleavage is at a β site by the β-secretase (BACE1) to release the large APP extracellular domain [1]. The remaining 99-amino acid C-terminal fragment is then cleaved at a variable γ-cleavage site within the transmembrane domain by the γ-secretase complex, releasing Aβ peptides of sizes ranging from 38 to 43 amino acids [2, 3].

Many studies have documented that the cleavage of APP into Aβ occurs after its endocytosis from the cell surface into the endosomal/lysosomal system [4]. Aβ production can be increased or reduced by manipulating APP re-internalization [5–7] and Aβ production is reduced by de-acidification of the endosomal-lysosomal system [8, 9]. The rapid dynamics of APP internalization and Aβ secretion suggest that early endosomes are an important site of processing of APP. However, other compartments have also been implicated including the ER [10–12], Golgi apparatus [13, 14] and the secretory pathway [15, 16] and currently there is no consensus as to the subcellular compartments responsible for Aβ production.

Work in our laboratory and others have suggested that the lysosome might also be a site of Aβ production. APP and γ-secretase proteins are highly enriched in purified lysosomes and in lysosome-related autophagosomes and phagosomes [17–20]. In the presence of protease inhibitors or in presenilin-1 (a component of the γ-secretase complex) knockouts, which lack γ-secretase activity, C-terminal fragments of APP accumulate in lysosomes [21, 22]. Moreover, Aβ is secreted in exosomes, which are intraluminal vesicles released from the endosomal/lysosomal system [9, 23]. We have recently shown, using APP fused to photoactivatable-GFP, that APP can also transit rapidly from the Golgi apparatus to the lysosome, where it is cleared by enzymes that are sensitive to disrupting lysosomal pH with chloroquine and to the γ-secretase inhibitor L685,485, suggesting that β- and γ-secretases function in this compartment. Furthermore, reducing lysosomal transport by knock down of the adaptor protein AP-3 reduces Aβ production by about one third [24].

Recently, we demonstrated a novel pathway, by which wild type APP selectively transits directly from the cell surface to lysosomes, bypassing the early and late endosomes [25]. Here, we demonstrate that in this pathway APP is transported into lysosomes *via* >500 nm macropinosome-like structures. These macropinosome-like structures endocytose the fluid-phase marker dextran. This process is inhibited by latrunculin B (which disrupts actin polymerization) and by depleting Rac1, but is enhanced by cell surface antibody binding of APP. We find that a dominant negative mutant of Arf6, a regulator of macropinocytosis, inhibits APP transit to the lysosome, but not to the endosome. Arf6DN decreases Aβ production >30 %, and this effect is similar in magnitude to blocking APP transport to early endosomes by a Rab5-dominant negative construct.

## Results

### Live cell imaging of SN56 cells shows rapid endocytosis of surface labeled APP to LAMP1 positive lysosomes via a large intermediate compartment

We have previously demonstrated the use of constructs to track the internalization of APP that consist of an N-terminal HA epitope tag, the C-terminal 112 amino acids of APP and a C-terminal Cyan Fluorescent Protein tag (ECFP) [25]. A linker next to the HA-tag also contains an optimized tetracysteine sequence for binding biarsenical fluorophores (FlAsH labeling) [26, 27]. These constructs have the same intracellular distribution and trafficking pattern as full-length APP [25, 24] and are referred to as HA-βAPP-CFP. To confirm our findings, we repeated key experiments with full-length (untagged) APP695.

Experiments were performed in primary mouse neurons or in the SN56 cell line, a hybrid cell line generated by fusing dissociated embryonic mouse septal neurons with N18TG2 neuroblastoma cells. Importantly, SN56 cells possess a neuronal morphology and cholinergic phenotype when differentiated and express endogenous APP [28–30].

**Fig. 1:**
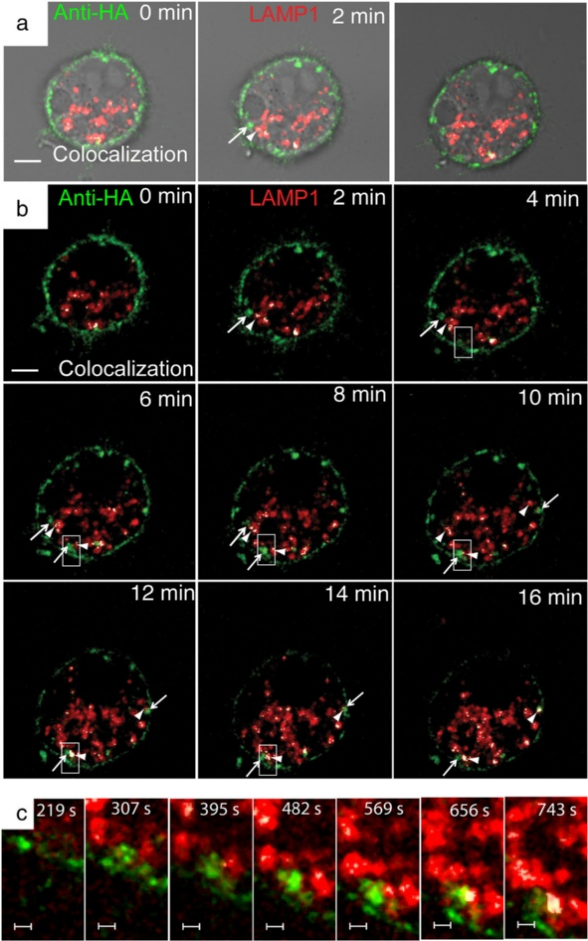
Live cell imaging demonstrates rapid transport of APP to Lysosomes. SN56 cells transfected with LAMP1-mRFP (red) and HA-βAPP-CFP (not shown) were surface labeled with Zenon-647 anti-HA antibodies (green) for 30 min, and then placed on a microscope stage at 37 °C and imaged by confocal microscopy. Images were taken about every 30 s. **a** Images showing a DIC brightfield channel superimposed on confocal microscopy images. Co-localized pixels were identified by Imaris software and are overlaid in white (see Methods). **b** Images showing confocal channels and colocalization channels without the brightfield channel. Surface labeled APP can be seen internalizing in very large vesicles (indicated by arrows) that fuse directly with lysosomes (arrow heads). Rectangle shows region enlarged in part **c**. (See also Additional file 2: Video S1A and B). Scale bar = 5 microns. **c** Enlarged inset of a single vesicle forming and fusing with a LAMP1 positive lysosome. Scale Bar = 1 micron

To visualize and track APP internalization into lysosomes in live SN56 cells, cells were transiently co-transfected with HA-βAPP-CFP and the Lysosomal Associated Membrane Protein 1 (LAMP1) fused to monomeric Red fluorescent Protein (mRFP). The next day, cells were surface labeled with Alexa Fluor 647-labeled anti-HA antibodies (generated using a Zenon labeling kit, Life Technologies) and then transferred to a heated microscope stage for immediate imaging. Labeling APP on the cell surface by means of an antibody is a standard technique used by many laboratories [31–35]. To identify and quantitate co-localized pixels, we used Imaris 7.6.4 software (Bitplane). Thresholds were set to identify the brightest 2 % of pixels in the green channel (surface labeled APP), and the brightest pixels in the red channel that would clearly demarcate lysosomes (up 2 % of the brightest pixels), and these were used to generate a co-localization channel which is overlaid in white (See Materials and methods [25, 36]). An example of the co-localization results is shown in Additional file 1: Figure S1A. In these experiments, APP internalizes rapidly into very large vesicles (~1 μm), which are much too large to be classical transport vesicles, and which then fuse with LAMP1 positive lysosomes (Fig. 1a, b, c and Additional file 2: Video S1A and B).

To rule out the possibility that LAMP1-mRFP overexpression might label other compartments in addition to lysosomes, and might also alter APP trafficking, we repeated these experiments in SN56 cells, identifying lysosomes using by immunostaining the endogenous marker LAMP2. Cells were transfected with HA-βAPP-CFP, labeled on ice with Alexa Fluor 647-labeled anti-HA antibodies and then either fixed immediately or allowed to internalize at 37 °C for 15 min before being fixed and imaged. Under these conditions, APP traffics to LAMP2-labeled lysosomes (Additional file 1: Figure S1B). To verify that the APP trafficking observed was not due to the use of modified APP constructs, the presence of fluorescent protein tags, or the presence of an HA tag, these experiments were repeated in cells transfected with a construct expressing full length APP 695, performing cell surface labeling using the anti-amyloid antibody 6E10 instead of anti-HA antibodies (Additional file 1: Figure S1B). In these experiments, full length APP is clearly transported to compartments labeled with LAMP1-mRFP (not shown) or immunostained endogenous LAMP2. In addition, Additional file 3: Figure S2 demonstrates that the surface labeled anti-HA-antibody binds the N-terminal of APP is migrating together with the C-terminal of APP labeled with a fluorescent protein tag.

Finally, to demonstrate that surface labeled APP is clearly internalized into lysosomes and not above or below them, we generated Z-stacks and performed 3D reconstruction of primary mouse cortical neurons that had internalized surface labeled (untagged) APP695 is colocalized with LAMP-mRFP labeled lysosomes (Additional file 4: Video S2 and Additional file 5: Video S3).

### APP is co-transported to lysosomes along with dextran, and this process is governed by actin, Rac1 and colocalizes with CTBp1/BARS

**Fig. 2:**
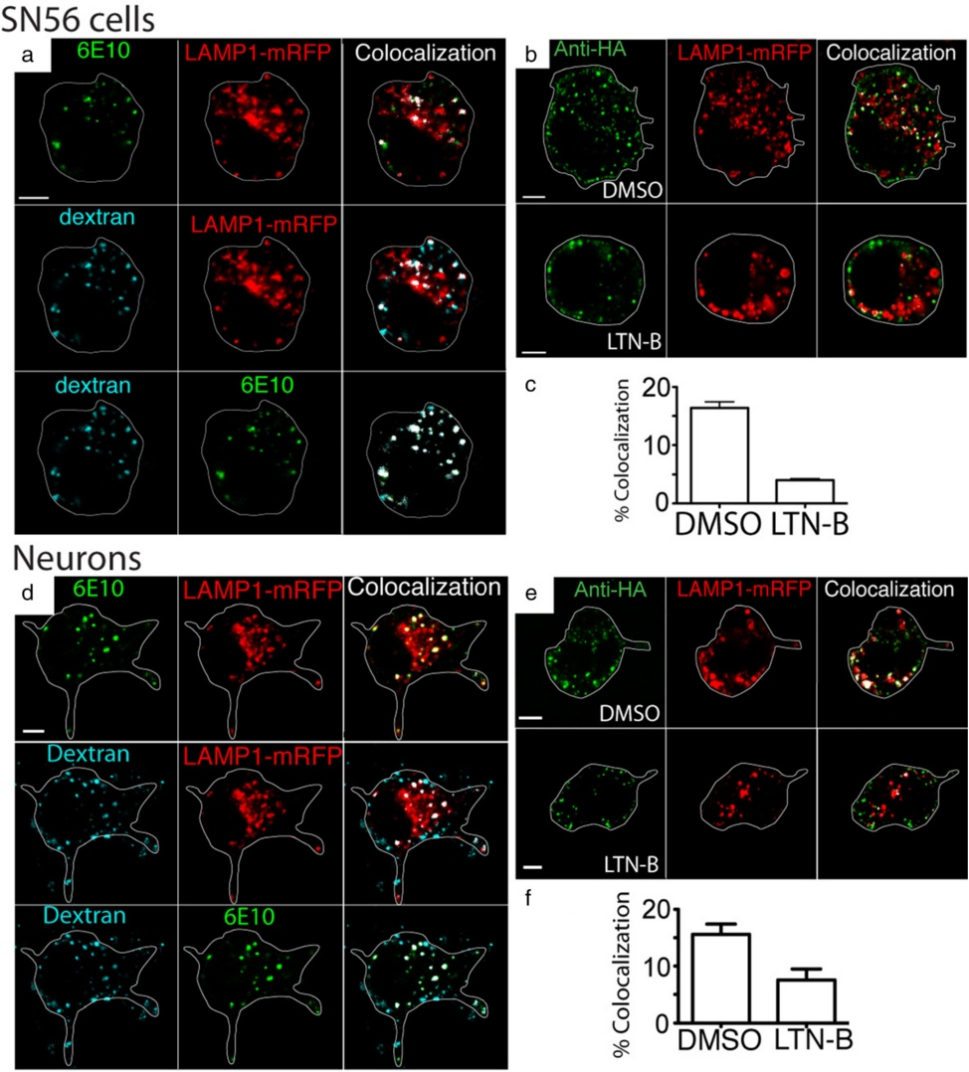
Rapid lysosomal transport of APP occurs by Macropinocytosis. **a** SN56 cells were transfected with untagged full length APP695 and Lamp1-mRFP (red) and surface labeled the antibody 6E10 labeled with Alexa Fluor 647 (green) on ice. Cells were placed in HBSS containing fluorescent dextran (blue) and allowed to internalize at 37 °C for 15 min, washed in HBSS and fixed. The approximate outline of each cell is superimposed for clarity. Colocalized pixels identified by Imaris software are overlayed in white. These images show that Surface labeled APP and dextran are both rapidly co-internalized together to a LAMP1 positive compartment. Scale bar = 5 microns. **b** SN56 cells were transfected with HA-βAPP-CFP and Lamp1-mRFP (red), surface labeled with Alexa Fluor 647 anti-HA antibodies (green) on ice and incubated at 37 °C for 15 min with or without 2.5 μM latrunculin B. **c** The percentage of green pixels (internalized surface-labeled APP) colocalized with red pixels (LAMP1) was calculated by Imaris Software and from at least 38 cells drawn from 3 separate experiments is graphed. * denotes *p* < 0.05 (*t*-test). **d** Mouse cortical neurons were transfected with untagged full length APP695 and Lamp1-mRFP (red) and surface labeled the antibody 6E10 labeled with Alexa Fluor 647 (green) on ice and then placed in HBSS containing fluorescent dextran (blue) at 37 °C for 15 min. Colocalized pixels are overlayed in white. APP and dextran are both rapidly internalize together to a LAMP1 positive compartment. Scale bar = 5 microns. **e** SN56 cells were transfected with HA-βAPP-CFP and Lamp1-mRFP (red), surface labeled with Alexa Fluor 647 anti-HA antibodies (green) on ice and incubated at 37 °C for 15 min with or without 2.5 μM latrunculin B. **f** Quantitation of the colocalization of internalized surface-labeled APP and LAMP1 was generated using Imaris software from at least 30 cells drawn from 4 independent experiments. * denotes *p* < 0.05 (*t*-test)

The large size diameter of the APP vesicles (Fig. 1a, b, Additional file 2: Video S1A and B) is reminiscent of macropinosomes. Furthermore, macropinosomes are known to rapidly fuse with lysosomes after internalization from the cell surface [37]. Macropinosomes are characterized by their ability up take up fluid phase markers, including dextran, and be dependent upon actin polymerization. We have previously demonstrated that in the absence of antibody-labeled APP on the cell surface, dextran was taken up first into endosomes, then into lysosomes, arriving in the lysosome after 30 min to an hour [25]. We repeated our internalization experiment, transfecting SN56 cells with APP695 and LAMP1-mRFP, surface labeled APP with Alexa Fluor 647 for 30 min on ice, and then placed the cells at 37 °C for 15 min with the addition of fluorescent dextran to the media, then washed and fixed the cells. In these experiments (Fig. 2a) both the antibody 6E10 and fluorescent dextran can be seen co-localized with LAMP1. This transport occurred much faster than the uptake of dextran to the lysosome without the cell surface antibody lableing, which we have previously observed [25]. We then repeated the internalization experiment after treating cells with latrunculin-B, which blocks macropinocytosis by disrupting actin filament formation [38]. Latrunculin-B reduced the co-localization of the surface-labeled APP with LAMP1-mRFP from 16.39 ± 1.05 % (DMSO alone) to 4.0 ± 0.25 % (*p* < 0.05) (Fig. 2b and c).

The findings above were then confirmed in mouse cortical neurons, where we found that surface labeled APP and fluorescently labeled dextran are also well co-localized with LAMP1 (Fig. 2d). As with SN56 cells, latrunculin-B also reduced the co-localization of the surface-labeled APP from 20.8 ± 2.98 % (DMSO alone) to 7.973 ± 1.025 in neurons (*t*-test) *p* < 0.05.

**Fig. 3:**
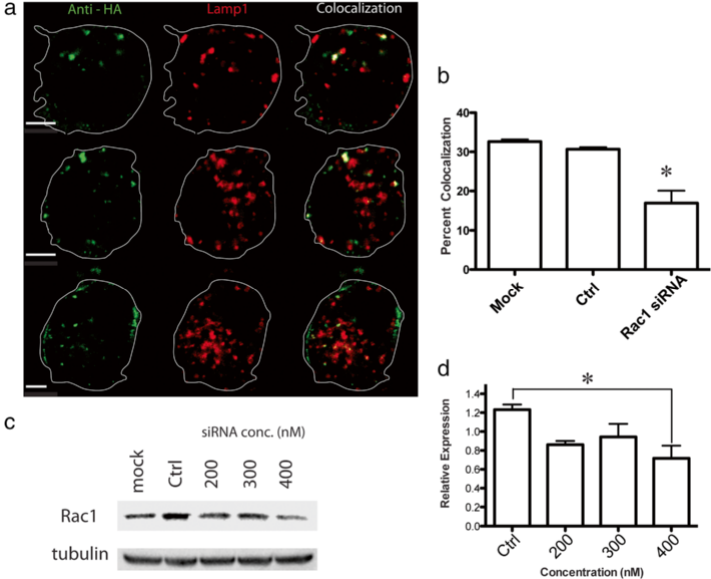
Rac1 knockdown with siRNA blocks direct lysosomal transport of APP. **a** SN56 cells were transfected with HA-βAPP-CFP (not shown) and LAMP1-mRFP (red), and 400 nM siRNA against Rac1. Cells were surface labeled on ice for with 647-labeled Anti-HA antibodies for 30 min and allowed to internalize APP for 15 min at 37 °C. Dye-labeled negative control siRNA was included with these transfections to confirm that they were transfected with siRNA (not shown). The approximate outline of each cell is superimposed for clarity. Co-localized pixels are shown in white. Scale bar = 5 microns. **b** Co-localization of internalized APP and LAMP1 in the presence of Rac1 siRNA was quantitated in at least 38 cells from at drawn from at least 4 experiments using a one-way ANOVA with Tukey post-test * denotes *p* < 0.05. **c** SN56 cells were grown in 60 mm dishes and transfected with increasing amounts of siRNA directed against Rac1. Cells were lysed and western blotted with antibodies against Rac1, stripped and reprobed with antibodies against tubulin. **d** Western blots from at least 3 experiments were quantified using ImageJ, and normalized to tubulin expression in 4 experiments using a one-way ANOVA with Tukey post-test. * denotes *p* < 0.05

Rac1 has been suggested as a critical protein for some forms of macropinocytosis [39]. Rac1 has been reported to be activated with Arf6 by the APP binding protein Fe65 [40]. In addition, Rac1 inhibition is also known to reduce Aβ production [41]. Therefore, we tested the ability of Rac1 depletion to block APP transport to lysosomes. To do this, we co-transfected Rac1 siRNA (400 nM), HA-βAPP-CFP, and LAMP1-mRFP, labeled cells on ice with Alexa Fluor 647-labeled anti-HA antibodies and then either fixed cells immediately or allowed them to internalize at 37 °C for 15 min before being fixed and imaged (Fig. 3). In these experiments, Rac1 siRNA reduced HA-APP co-localization with LAMP1 from 30.7 % ± 0.49 % to 16.9 3.16 % (*p* < 0.05) (Mean ± SEM). The involvement of Rac1 with APP internalization supports the idea that APP is undergoing macropinocytosis.

**Fig. 4:**
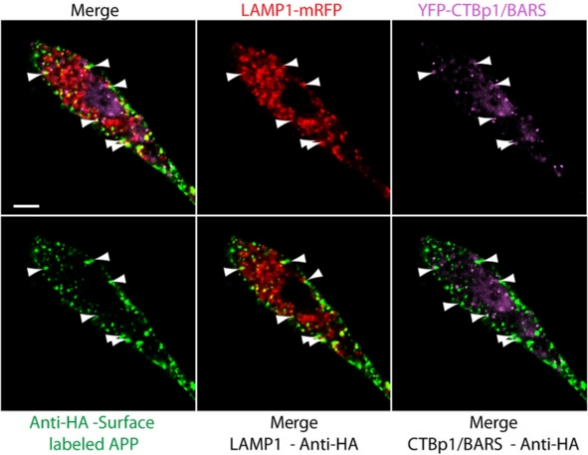
CTBp1/BARS is colocalized with Anti-HA and LAMP1-mRFP in macropinosomes at the cell surface. SN56 cells transfected with LAMP1-mRFP (red) and HA-βAPP-CFP (not shown), and CTBp1/BARS-YFP (purple) were surface labeled with Zenon-647 anti-HA antibodies (green) for 30 min, incubated at 37 °C for 15 min, fixed and imaged. Colocalization of surface labled APP, CTBp1/BARS, and LAMP1 appears in structures near the cell surface. Scale bar = 5 microns

The C-terminal–binding protein-1 short-form/BFA–ADP-ribosylation substrate (CtBp1/BARS) is a dual function protein, known to act as a transcription factor in the nucleus, but also as a regulator of membrane fission [42]. CTBp1/Bars has been shown to localize to the macropinocytic cup, where it functions as a motor to promote dynamin-independent membrane fission, detaching newly formed macropinosomes from the plasma membrane [43]. To examine this protein in relation to APP internalization, we transfected SN56 cells with APP-CFP, LAMP1-mRFP and CTBp1/BARS-YFP. Cells were then surface labeled with Alexa Fluor-647 labeled anti-HA on ice for 15 min, allowed to internalize for 15 min at 37 °C and then fixed and imaged. As predicted, CTBp1/BARS-YFP is visible colocalized on only a minority of vesicles that are positive for anti-HA and LAMP1, with a predilection for vesicles at the cell surface (Fig. 4). The colocalization of surface-labeled APP with CTBp1/BARS suggests that APP is being taken up by macropinosomes.

### Electron microscopy demonstrate that cell-surface labeled APP is internalized in macropinosomes

**Fig. 5:**
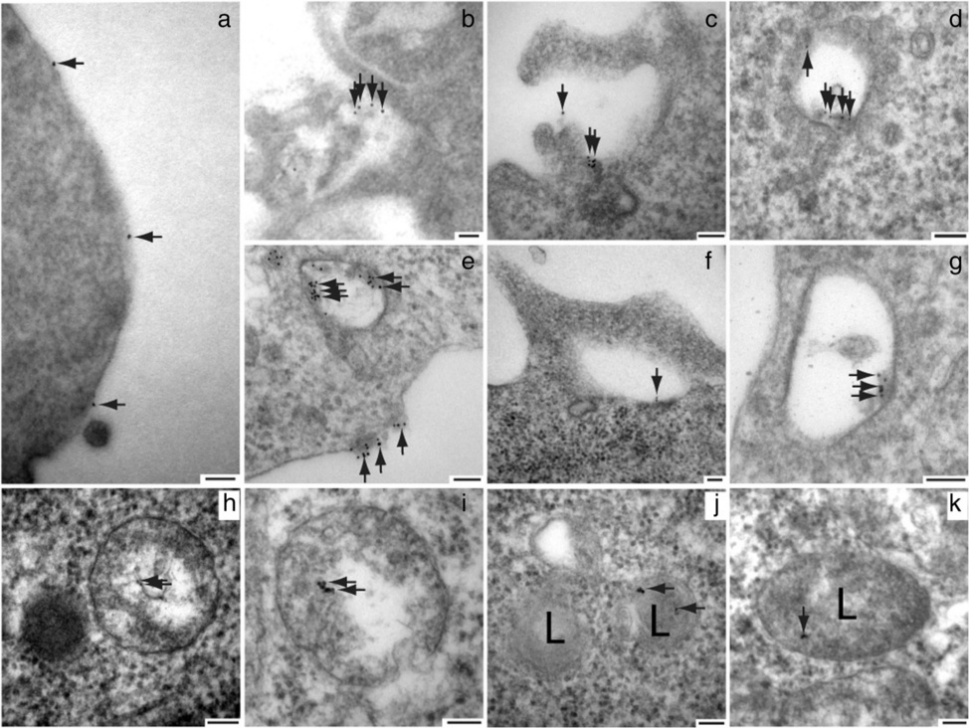
Electron Microscopy of APP Endocytosed into Macropinosomes and Lysosomes. SN56 cells were transfected with HA-βAPP-CFP and surface-labeled with Anti-HA antibody, followed by an anti-mouse antibody labeled with 10 nm gold and Alexa Fluor 488 on ice. When cells were fixed and sectioned immediately (**a**) gold particles are seen only on the cell surface. After incubation for 15 min at 37 °C, gold particles are seen collected at the cell surface in regions of membrane ruffling (**b**, **c**). Gold particles were seen in structures surrounded by vesicles and tubules suggestive of sorting endosomes (**d**) as well as in large clear structures with the appearance of macropinosomes (**e**, **f**, **g**). Gold particles were also seen in structures containing a mixture of clear areas and electron dense regions, suggestive of macropinosomes fused with primary lysosomes (**e**, **h**, **i**) and in electron dense structures typical of lysosomes (**j**, **k**). Scale Bar = 100 nm

To examine APP trafficking by electron microscopy, we repeated the surface labeling experiment using an anti-HA antibody and a secondary antibody conjugated to 10 nm gold particles. Cells were either fixed directly or allowed to internalize for 15 min at 37 °C and fixed, sectioned and examined using Transmission Electron Microscopy. At time 0, gold particles were seen scattered on the cell membrane (Fig. 5a). After incubation at 37 °C for 15 min., it was possible to observe gold particles collecting in large groups on the cell surface within regions of membrane ruffling (Fig. 5b, c). Some gold particles were seen in large organelles with tubules surrounded by very small (<100 nm) transport vesicles, suggestive of sorting endosomes (Fig. 5d). Gold particles were also seen in large (>500 nm) clear vesicles near the cell surface characteristic of macropinosomes (Fig. 5 e, f, g). Finally, gold particles were observed in large (>500 nm) hybrid organelles with clear and electron dense regions suggestive of a macropinosome fused with a primary lysosome (Fig. 5 e, h, i) and within structures with the classic electron dense appearance of lysosomes (Fig. 5j, k). These images are strikingly similar to previous work examining macropinocytosis by electron microscopy [37, 44].

### Internalization of APP into lysosomes is enhanced by cell surface binding/crosslinking of APP

**Fig. 6:**
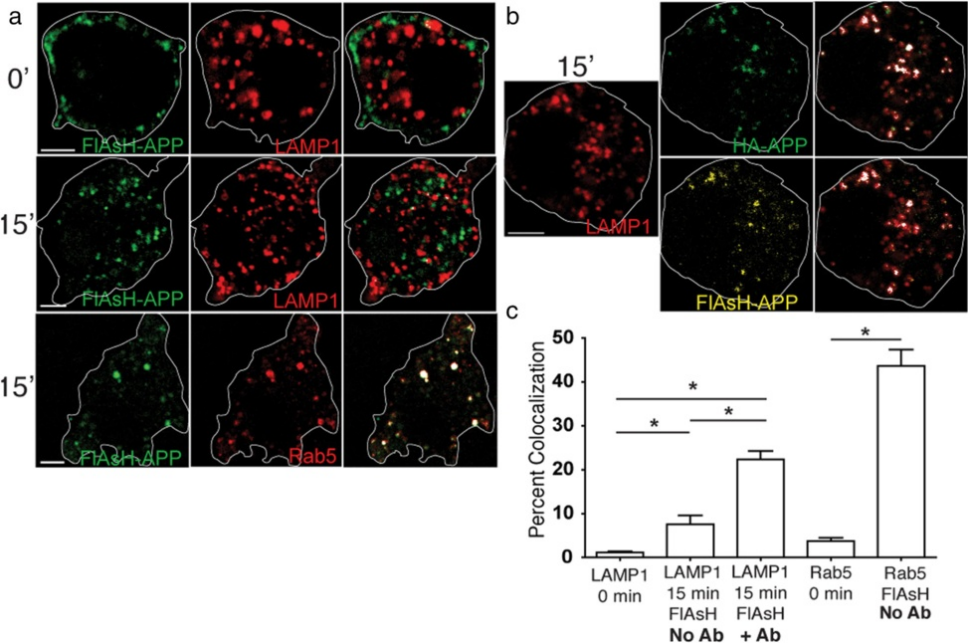
Cell surface antibody binding enhances rapid transport of APP to the lysosome but not to the early endosome. **a** SN56 cells were transfected with βAPP-CFP (not shown) and either LAMP1-mRFP or Rab5-mRFP or (red). APP was surface-labeled with FlAsH reagent (green) on ice for 3 min. No antibody labeling was used. Cells were either directly fixed (0 min) or incubated at 37 °C for 15 min and then fixed. Co-localized green/red pixels are overlayed in white. Each image shows a single cell, with approximate outline superimposed for clarity. In the absence of antibody, FlAsH labeled APP moves mostly to the early endosome. Approximate outlines of each cell is overlaid. Scale bar = 5 microns. **b** SN56 cells were transfected with HA-βAPP-CFP (not shown) and LAMP1-mRFP (red). APP was surface-labeled with FlAsH reagent (yellow) on ice for 3 min and then with 647-labeled anti-HA antibody (green) for 30 min on ice. After 15 min at 37 °C, both labels show extensive co-localization with lysosome. **c** Co-localized pixels were quantitated from the antibody crosslinking experiments in A and B in at least 10 cells from at least 3 experiments using an ANOVA with Tukey post-test. * denotes *p* < 0.05. Scale bar = 5 microns

Macropinocytosis can occur constitutively, but is often triggered by cell surface receptor binding [39, 45, 46]. Similarly, the internalization of APP can be triggered by antibody binding/crosslinking at the cell surface [34, 35]. In order to determine if lysosomal transport of APP is influenced by cell surface antibody binding, we surface labeled HA-βAPP-CFP with a biarsenical-fluorescein reagent (FlAsH), which binds to the engineered N-terminal tetracysteine FlAsH tag in the construct. This labeling technique has been previously used to selectively label cell surface proteins [47] (Fig. 6a). In the absence of antibody, there was robust co-localization of surface labeled APP with Rab5-labeled early endosomes (43.7 ± 3.7 %) at 15 min, whereas only 7.6 ± 2.1 % of this fluorescence was co-localized with LAMP1. When FlAsH-labeled cells were then further surface-labeled with anti-HA antibodies, the amount of APP translocating to the lysosome tripled to 22.4 ± 1.9 % (Fig. 6b and c) (*p* < 0.05). We also found increased APP trafficking to lysosomes with increasing antibody concentration (Additional file 6: Figure S3). Therefore, in the absence of antibody binding, cell surface APP traffics primarily to early endosomes, although some moves constitutively to lysosomes. However cell surface antibody binding/crosslinking stimulates lysosomal trafficking of APP. This suggests that APP may play a role as a cell surface receptor.

### Arf6 mediates APP trafficking to lysosomes but not endosomes

**Fig. 7:**
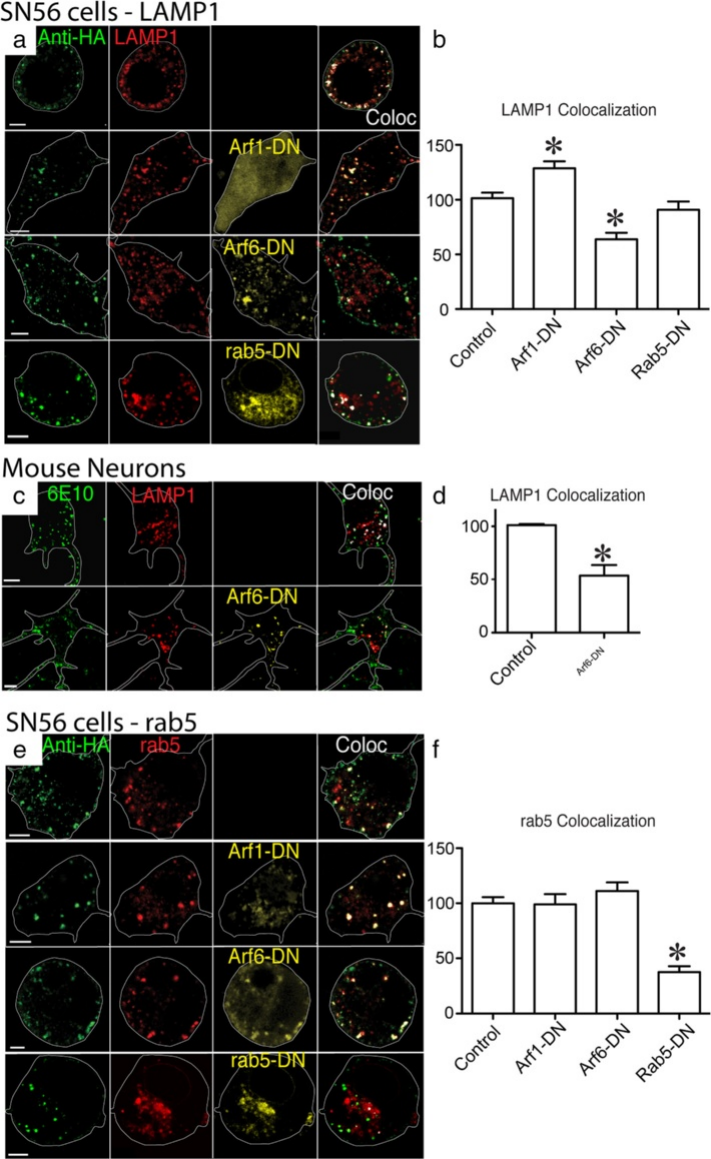
An Arf6-DN mutant blocks APP transport to the lysosome but not the early endosome. **a** SN56 cells were transfected with HA-βAPP-CFP (not shown), LAMP1-mRFP (red) and a dominant negative mutant of a GTPase fused to fluorescent protein (yellow or cyan) as indicated. Cells were then surface-labeled with 647-labeled anti-HA antibody (green) for 30 min on ice and then allowed to internalize APP for 15 min at 37 °C and fixed. Each panel shows a single cell with approximate its outline superimposed for clarity. Merged red and green channels are shown, with co-localized pixels overlayed in white. Cell outlines are overlaid in white. Scale bar = 5 microns. **c** Quantitation of APP internalization to the lysosome with values normalized to HA-βAPP-CFP/LAMP1-mRFP cells examined at the time of the experiment. Colocalization was determined from more than 37 cells drawn from at least 4 transfections, and analyzed by a one way ANOVA with Tukey Post-test. * denotes *p* < 0.05. **b** Mouse cortical neurons were transfected with APP695, and LAMP1-mRFP (red), with or without Arf6-DN. Cells were then surface-labeled with 647-labeled 6E10 (green) and then allowed to internalize. Merged red and green channels are shown. Co-localized pixels overlayed in white. Scale bar = 5 microns. **d** Quantitation of APP internalization to the lysosome normalized to lysosome with values normalized to APP695/LAMP1-mRFP transfected cells at the time of the experiment. Data is from at least 58 cells in each group drawn from 5 experiments using a one-way ANOVA with Tukey post-test. * denotes *p* < 0.05. **e** SN56 cells were transfected with HA-βAPP-CFP (not shown), rab5-mRFP (red) and a dominant negative mutant of a GTPase as indicated. Merged red and green channels are shown, with co-localized pixels overlayed in white. Scale bar = 5 microns. **f** Quantitation of APP internalization to the early endosome, with values normalized to HA-βAPP-CFP/rab5-mRFP cells examined at the time of the experiment. Data is from more the 30 cells drawn from at least 4 transfections using one-way ANOVA with Tukey post-test. * denotes *p* < 0.05

The GTPases Arf1 and Arf6 have been reported as regulating clathrin independent internalization [48, 49]. We examined the effects of these proteins on lysosomal transport in SN56 cells, which were transiently transfected with HA-βAPP-CFP LAMP1-mRFP, and the regulatory proteins Arf1, Arf6 or Rab5 containing a dominant negative mutation. These cells were then allowed to internalize at 37 °C. for 15 min. The extent of co-localization was reported as a percentage of control levels to account for inter-experimental variability. Surface labeled APP co-localized with LAMP1 100 ± 4.8 % and this was increased by a dominant-negative Arf1-T31N-DN (128.6 ± 6.3 %; *p* < 0.05), and significantly reduced by dominant-negative Arf6-T27N-DN (63.75 ± 5.2 %; *p* < 0.05). Internalization to the lysosome was not significantly affected by a dominant negative Rab5-S34N-DN (90.8 ± 7.6 %) (Fig. 7a and b). We repeated this experiment in primary mouse neurons, where we again found that the transfection of Arf6-T27N-DN reduced uptake of APP to the lysosome from 100 ± 1.1 % to 54.9 ± 9.8 % (*p* < 0.05) (Fig. 7b and c).

We then investigated the effects of Arf1, Arf6, and Rab5 dominant negative mutations on the internalization of APP to early endosomes labeled with Rab5-mRFP (Fig. 7c). In these experiments, surface labeled APP was co-localized with endosomal marker Rab5 (100 ± 5.25 %) and this transport was significantly reduced by Rab5-S34-DN, (37.7 ± 5.2 %; *p* < 0.05) (Fig. 7d) but was unaffected by Arf1-T31N-DN and Arf6-T27N-DN at 98.9 ± 9.37 % and 111.2 ± 37.7 % respectively) (Fig. 7 e and f).

**Fig. 8:**
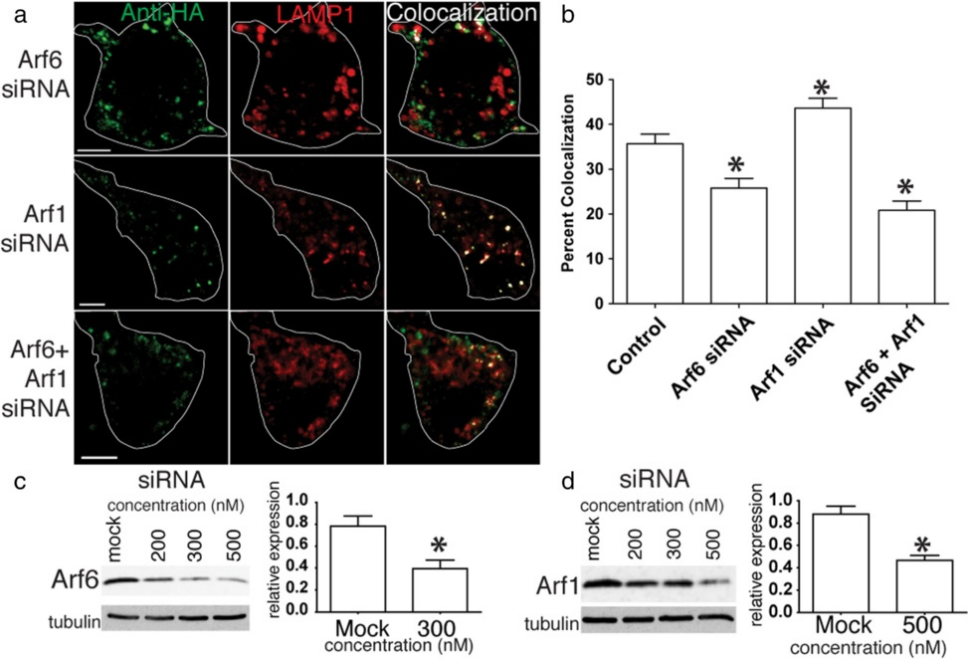
Arf6 and Arf1 knockdown with siRNA also blocks direct lysosomal transport of APP. **a** SN56 cells were transfected with HA-βAPP-CFP (not shown) and LAMP1-mRFP (red), and siRNAs against Arf6 and Arf1. Cells were surface labeled on ice for with 647-labeled Anti-HA antibodies for 30 min and allowed to internalize APP for 15 min at 37 °C. Co-localized pixels are shown in white. Scale bar = 5 microns. The approximate outline of each cell is superimposed for clarity. **b** Co-localization of internalized APP and LAMP1 in the presence of ARF6 and Arf1 siRNAs was quantitated in at least 30 cells from at drawn from at least 3 experiments. * denotes *p* < 0.05. **c** Western blots demonstrating Arf6 and Arf1 are knocked down with increasing amounts of siRNA. **d**) were quantitated in from at least 3 experiments. These are normalized to tubulin expression and quantified

We confirmed the effects of the Arf1 and Arf6-DN mutants using siRNA knockdown. In these experiments the Arf6 siRNA significantly reduced APP co-localization with LAMP1 from 35.7 ± 2.1 % (control) to 25.8 ± 2.2 %, while the Arf1 siRNA significantly increased APP co-localization to 43.6 ± 2.2 % (Fig. 8) (*p* < 0.05). When combined, the Arf6 and Arf1 siRNAs together reduced APP internalization to 20.9 ± 2.0 % (Fig. 8). These results demonstrate that Arf6 is required for rapid transport of APP to the lysosome.

**Fig. 9:**
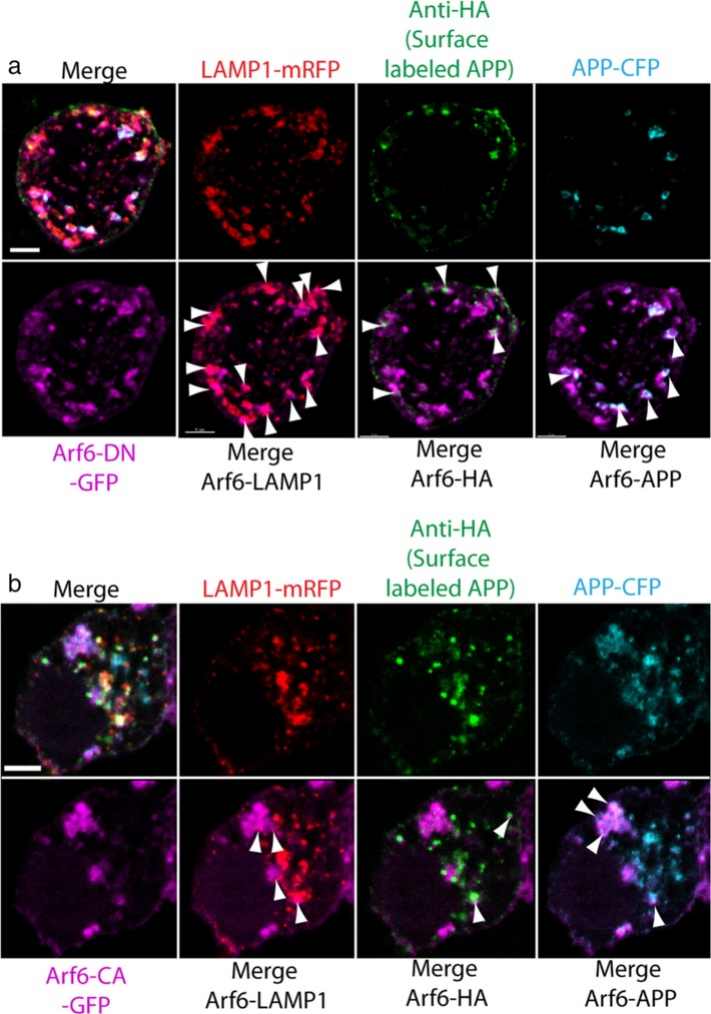
An Arf6-DN mutant blocks is localized at the cell surface and on lysosomes. SN56 cells were transfected with HA-βAPP-CFP, LAMP1-mRFP, and Arf6 bearing either a dominant negative GFP-Arf6-T27N-DN or constitutively active GFP-Arf6-Q67L-CA mutation. Cells were surface labeled Alexa Fluor 647 labeled anti-HA antibodies on ice and then allowed to internalize at 37 °C and imaged by confocal microscopy. **a** Images show the individual color channels of LAMP1, HA (surface-labeled APP), and CFP (APP) along the top row. On the bottom row is the distribution of GFP-Arf6-DN by itself, and merged each of the above channeled. Arf6-DN protein appears on the cell surface and in large intracellular organelles, colocalized with LAMP1 (arrowheads), surface-labeled HA-APP (arrowheads) and with the CFP tag on APP (arrowheads). **b** The constitutively active GFP-Arf6-Q67L-CA, is also present at the cell surface, the cytosol, and on many intracellular organelles. It demonstrates on a much less localization with LAMP1 (arrowheads), surface-labeled HA-APP (arrowheads) or the CFP tag on APP (arrowheads)

We examined the distribution of Arf6 in NS56 cells. SN56 cells were transfected with HA-βAPP-CFP, LAMP1-mRFP, and Arf6 bearing either a dominant negative GFP-Arf6-T27N-DN or constitutively active GFP-Arf6-Q67L-CA mutation (Fig. 9). Cells were surface labeled Alexa Fluor 647 labeled anti-HA antibodies on ice and then allowed to internalize at 37 °C and imaged by confocal microscopy. In these images the Arf6-DN protein appears on the cell surface along with surface-labeled HA-APP label indicating that these cells not internalizing APP well. In addition, GFP-Arf6-T27N-DN appears in large intracellular organelles, where it is localized with LAMP1-mRFP and with CFP tag on APP (that did not need to transit to the cell surface for labeling). In contrast, the constitutively active GFP-Arf6-Q67L-CA, while also residing at the cell surface, was present throughout the cytosol. It also labeled many intracellular organelles, only a portion of which were also LAMP1-mRFP or APP-CFP positive. These results are similar to what has been described for Arf6 localization [50].

**Fig. 10:**
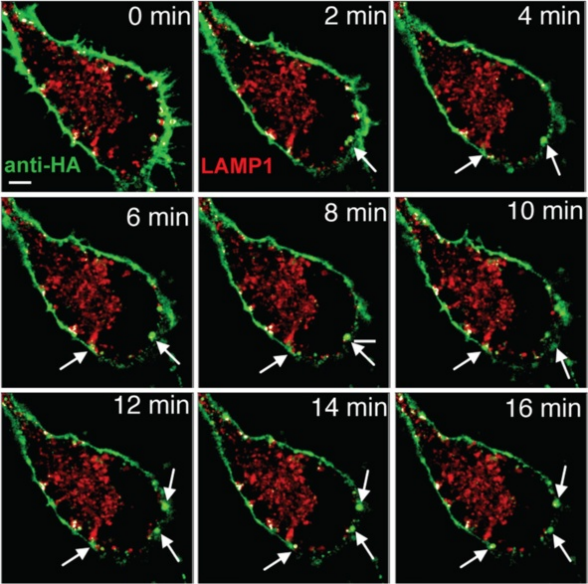
Arf6-DN arrests APP internalization at the cell surface. SN56 cells transfected with HA-βAPP-CFP (not shown) and LAMp1-mRFP (red) and were surface labeled with Zenon-647 anti-HA antibodies (green) for 30 min. Cells were placed on a microscope stage at 37 °C and imaged by confocal microscopy every 30 s. Co-localized red and green pixels are overlayed in white. Surface labeled APP can be seen entering large vesicles that fail to fuse with lysosomes. (See also Additional file 7: Video S4). Scale bar = 5 microns

To confirm the effects of the Arf6-DN mutant in live cells, we repeated the APP internalization experiment with Arf6-DN in live (unfixed) cells. SN56 cells were transfected with HA-βAPP-CFP LAMP1-mRFP, and Arf6-DN, surface labeled with Alexa Fluor 647 anti-HA on ice, and then placed on the stage of a confocal microscope and imaged over time. In these experiments, APP appears to collect in large structures that remain at the cell surface, which fail to migrate inward to fuse with LAMP1-mRFP labeled lysosomes suggesting a failure of fission and/or transport. (Fig. 10 and Additional file 7: Video S4).

### Arf6 expression in human hippocampus parallels the development of Alzheimer’s disease

**Fig. 11:**
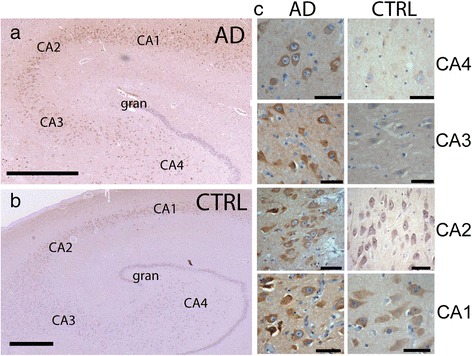
Arf6 expression spreads through the Hippocampus with progression of Alzheimer’s disease. Hippocampal sections from Alzheimer’s disease and Control subjects were stained with an antibody to Arf6. In low power images images (Aperio Slide scanner), Arf6 can be seen darkly staining CA1-CA4 pyramidal cells in AD brain (**a**) but only CA1 and CA2 in Control sample (**b**) Hippocampal Granule cells (gran) are only lightly stained. Scale Bar 1000 μm. **Panel c** shows high power (40X) images of representative pyramidal cells from each hippocampal region (scale bar 50 μm)

Given the importance of Arf6 in mediating APP internalization into lysosomes, we sought to determine if Arf6 expression was altered in AD. We chose to study expression in the hippocampus, which is invariably affected in AD, with pathological changes including neurofibrillary tangles beginning in the subiculum/CA1 fields, and then progressing through the pyramidal cell fields to finally affect CA4 neurons Braak *et al.* [51]. We performed immunostaining of Arf6 in the human hippocampus of individuals with neuropathologically confirmed AD (n = 8, average age 75, range 62 - 89). These individuals were compared with a control group containing individuals that did not have a pathological diagnosis of Alzheimer’s disease, although some had mild senile changes (n = 5, average age 69.5, range 51 – 79). Arf6 staining, in controls and AD patients, follows a laminar pattern and preferentially stains large pyramidal neurons of CA1 and CA2 fields of the hippocampus. These pyramidal neurons are also preferentially afflicted by neurofibrillary tangles in AD [51]. Interestingly, 7 of the 8 AD individuals also exhibited intense staining of CA3 and CA4 pyramidal neurons, which was not present in the controls (Fig. 11). These observations suggest that increased Arf6 expression increases in AD and follows the pathological progression of AD through the hippocampus.

### The Arf6-DN mutant decreases Aβ production by blocking lysosomal transport

**Fig. 12:**
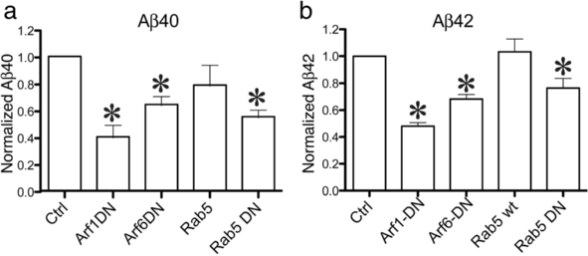
Arf6 and Arf1 Dominant negative mutations reduce Aβ production. SN56 cells were transfected with HA- βAPP-CFP, a construct bearing rab5, Arf1 or Arf6 as indicated. After 3 days, media was assayed using an Aβ42 or Aβ40 ELISA. Results are from 4-8 independent experiments with assays performed in duplicate or triplicate, and normalized to control cells. Analysis was performed using a one-way ANOVA with Tukey post-test. **a** Concentrations of Aβ40 normalized to control. **b** Concentrations of Aβ42 normalized to control. * denotes *p* < 0.05

We then characterized the effects of these mutant GTPases on APP processing into Aβ. Cells were transfected with HA-βAPP-CFP and media was collected and assayed for Aβ40 and Aβ42 secretion by ELISA. In these experiments, control cells produced 523.1 ± 77.96 pg/ml of Aβ40 and 104.2 ± 11.81 pg/ml of Aβ42. Results were normalized to HA-βAPP-CFP transfected cells and plotted in Fig. 12. We found that both Arf1-DN and Arf6-DN significantly reduced Aβ40 production to 40.3 ± 9.3 % and 64.4 ± 6.7 % respectively (*p* < 0.05). By comparison, transfection with Rab5-WT did not significantly reduce Aβ40 (78.9 ± 15.3 %) while DN-Rab5 reduced Aβ40 production to 55.2 ± 5.8 % (*p* < 0.05) (Fig. 12a). Similarly, we found that Aβ42 secretion was also significantly reduced by the Arf1-DN and Arf6-DN constructs to 48.1 ± 3.7 % and 68.2 ± 3.5 % of control respectively (*p* < 0.05). Again, Rab5-WT had no significant effect (100 ± 9.5 %) while the Rab5-DN reduced Aβ42 production to 76.5 ± 7.1 % (*p* < 0.05) (Fig. 12b). These experiments demonstrate that in un-stimulated cells, the Arf6-mediated direct lysosomal pathway is responsible for about one third of Aβ production, which is comparable to the amount of Aβ reduced by blocking the classical Rab5-mediated endocytic pathway.

## Discussion

Previous studies in our laboratory have shown that APP is transported by a novel, rapid trafficking pathway directly from the cell surface to the lysosome (Lorenzen *et al.* [25]) suggesting that the lysosome may be a site of APP processing. Here we demonstrate that APP is internalized rapidly to the lysosome from the plasma membrane *via* large clear structures (>500 nm) in a process consistent with macropinocytosis. This transport occurs in resting cells, and is enhanced by antibody binding to APP at the cell surface. An Arf6 dominant negative construct inhibited APP transit to the lysosome, but not to the endosome. Arf6 staining increases in the brains of AD patients and correlates with the progression of hippocampal involvement in AD, suggesting that Arf6 may be involved in AD pathology. Finally we show that reducing rapid transport of APP to the lysosome significantly decreases the amount of secreted Aβ40 and Aβ42 suggesting that this pathway accounts for about half of the Aβ produced from cell surface APP.

Observations from live cell internalization studies revealed that direct lysosomal trafficking of APP occurred in a compartment that appeared to be too large to be clathrin-dependent vesicles. Of the known endocytic pathways, only phagocytosis and macropinocytosis use the large vesicles observed here (0.2 μm - 1 μm) [39, 52]. In phagocytosis, large particles are engulfed by receptor-activated membrane extensions that adhere directly to the surface of the particle. In contrast, macropinocytosis results from the extension of membrane processes to engulf large volumes of extracellular fluid without the requirement of an extracellular particle [39, 52]. Macropinocytosis can occur constitutively, but is often stimulated by ligand binding [39]. Once formed, macropinosomes rapidly fuse with lysosomes within minutes [37, 44, 53].

Although commonly associated with immune cells, macropinocytosis has been reported in a very small number of papers in neurobiology. Macropinocytosis has been implicated in neurons for growth cone extension/collapse and bulk membrane retrieval [54, 55], internalization of the prion protein and misfolded SOD1 [56, 57] and the uptake of Aβ by macropinocytosis in astrocytes and macrophage [58, 59]. Although this is the first report of macropinocytosis of APP, the transport of APP to lysosomes within 15 min and to large clear structures resembling macropinosomes has been previously observed [32].

Antibody binding at the cell surface has been shown to trigger endocytosis and processing of APP in several previous studies [34, 35, 60, 61], which we show stimulates macropinocytosis. This is consistent with APP’s proposed role as a cell surface receptor where it’s processing is analogous to the Notch receptor [62, 63]. The Notch receptor is also cleaved on it’s extracellular/luminal domain before being cleaved by the γ-secretase, freeing its cytoplasmic C-terminal fragment to translocate to the nucleus and activate gene expression along with the adaptor protein Fe65 [64, 65].

Recently, Fe65 was shown to directly bind and activate Arf6, which also results in Rac1 activation [40] suggesting that these proteins play a role in APP processing. Rac1 inhibition itself has also been demonstrated to reduce Aβ production [41, 66]. Our work therefore provides a link between activation of Arf6/Rac1 and γ-cleavage, because activating these proteins causes internalization of cell surface APP to compartments that are able to perform the γ-cleavage.

Arf6 has been implicated in a wide variety of cellular functions including clathrin-dependent and independent internalization, endosomal sorting and recycling, actin remodeling [67, 68] and the generation of macropinosomes and autophagosomes [69, 70]. To our knowledge there is only one other report documenting a relationship between Arf6 and Aβ production [71]. In this study in HeLa cells, DN-Arf6-T27N increased Aβ secretion by increasing BACE internalization into clathrin-independent compartments that eventually fused with APP-containing endosomes. These results differ from ours because it was performed using APP with the Swedish mutation, which does not undergo rapid lysosomal transport [25]. It is also possible that Arf6 might function differently in neuronal cells compared to HeLa cells used there. In any case, these results are also different from the expected if Arf6 activates Rac1, which is known to increase Aβ production.

The effects of Arf1 are surprising in that the Arf1DN increases APP internalization to the lysosome and yet decreases Aβ production. Arf1 is thought to primarily regulate trafficking vesicles out of the Golgi apparatus [67], but can also play a role in clathrin independent macropinocytosis [48, 49, 72]. However, as its main proposed role is the formation of COP1 coated vesicles, which are critical for protein export from the Golgi apparatus, it is likely that the reduction in Aβ production seen in these experiments is related to the reduction of protein trafficking out of the Golgi apparatus.

Our data suggest that Aβ is being generated in the lysosome. Although lysosomes are usually regarded as degradative organelles, they could be the source of Aβ secreted in exosomes [9, 23]. The production of Aβ in lysosomes has broad implications for Alzheimer’s disease pathogenesis [73]. Aβ fibrilogenesis is nucleated by both lysosomal gangliosides and the acidic lysosomal pH of 4.5 [74, 75] and the lysosome may be the initial site of seeding of insoluble Aβ [4, 76, 77]. These aggregates can disrupt neuronal synapses [78] and membranes [79] leading to lysosomal rupture and cell death [80]. The intracellular/intralysosomal accumulation of Aβ42 has been demonstrated in many transgenic mice [77] and human neuropathological material before the appearance of plaques [81, 82]. The presence of active lysosomal enzymes in amyloid plaques also suggest that amyloid plaques may have a lysosomal origin [83]. Therefore, Aβ produced by macropinocytosis is introduced directly into an environment where it can potentially lead to pathology.

## Conclusion

In conclusion, this study demonstrates that APP is transported directly from the cell surface to lysosomes by a process termed macropinocytosis. This process is driven by cell surface antibody binding, reinforcing the idea that APP might function as a receptor for an as-yet undefined ligand. This process is mediated by Arf6 and the reduction of Arf6 activity significantly reduced the secretion of Aβ. This work suggests that the lysosome is a source of Aβ. We hope that a better understanding of APP trafficking may yield a promising therapeutic target for Alzheimer’s disease.

## Materials and methods

### Antibodies and reagents

Antibodies were: Mouse Anti-HA (Roche Applied Science), Rabbit Anti-Arf6 (Sigma), Rabbit Anti-Arf1 (Epitomics), Rabbit Anti-Rac1 (Santa Cruz Biotechnology). SN56 cells were obtained from Dr. Jane Rylett. Fluorescently-labeled secondary antibodies and Zenon® Alexa Fluor® 647 Mouse IgG2b Labeling Kit were purchased from Life Technologies (California) Fluorescent dextran (Cascade blue, 3000 Da, lysine fixable) was purchased from Life Technologies (California). ELISA assays were from Life Technologies (California). Dulbecco’s modified Eagle’s medium, fetal bovine serum, Hank’s balanced salt solution (HBSS), penicillin, streptomycin, trypsin and neurobasal media were all purchased from Gibco.

### DNA constructs

The βAPP construct has been previously demonstrated Lorenzen *et al.* [25]. This cDNA was generated from cDNA encoding APP 750- YFP (a gift of Dr. Bradley Hyman). Briefly, the signal sequence (including the required sequence for signal peptide cleavage) was cloned using the primers 5′GCTAGCATGCTGCCCGGTTTG3′ and 5′ACGCGTAGCGTAATCTGGAACATCGTATGGGTACTCCAGCGCCCGA3′, which add a 5′Nhe1 site and 3′ haemagglutinin (HA) tag and a 3′Mlu1 site. The C-terminal 112 amino acids beginning 12 amino acids upstream of the ®-cleavage site was cloned using the primers 3′ACGCGTTTCCTGAACTGCTGCCCCGGCTGCTGCATGGAGCCC5′ and 3′ATCAAGACGGAGGAGATCTCTG5′. These primers also add an optimized 5′-FlAsH tag Martin *et al.* [27] restriction site and 5′Sal1 site and a 3′Mlu1 site. These 2 products were ligated into pEYFP-N1 or pECFP-N1 vectors (Clontech).

Expression constructs for regulatory proteins bearing dominant negative mutations are GFP-Arf6-T27N and GFP-Arf6-Q61L (A generous gift of Dr. Julie Donaldson), YFP-CTBP1/BARS and (A generous gift of Dr. Alberto Luini), YFP-Arf1-T23N (A generous gift of Dr. Jean Gruenberg). LAMP1-YFP was a generous gift from Dr. Walter Mothes and recloned into mRFP.

### Cell culture and transfection

SN56 neuroblastoma cells were grown in Dulbecco’s modified Eagle’s medium (DMEM), respectively supplemented with 10 % (v/v) heat inactivated fetal bovine serum (FBS) (Gibco), and 100 μg/ml penicillin/streptomycin (Gibco). All cells were kept in an incubator at 37 °C in a humidified atmosphere containing 5 % CO2. Cells were kept in culture in 75 cm^2^ flasks (Falcon) and were split every 3-4 days. Cells were seeded at a density of 3 × 10^5^ cells/35-mm dish (MatTek) one day prior to being transfected. Cells were then transiently transfected using Lipofectamine 2000 following manufacturer’s instructions (Invitrogen) in serum free media. Following a 24 h incubation period, cells were differentiated before imaging by the addition of 1 mM dibutyryl cyclic AMP (dbcAMP; Sigma) to serum free medium Pedersen *et al.* [28]; Hammond *et al.* [29].

### Neuronal culture

Pregnant CD1 mice were obtained from Charles River under animal protocols approved by the University of Western Ontario Animal Care Committee. Primary prefrontal cortical neurons were prepared from E15 mouse embryos as described previously^1^. Neurons were seeded and grown in poly-L-ornithine coated plates and maintained in Neurobasal medium supplemented with 1X B27 and 0.8X N2 supplements, 2 mM glutamax and 50 U/ml penicillin/streptomycin (Invitrogen). Cells were grown at 37 °C in a humidified atmosphere containing 5 % CO2. One half of the volume of neurobasal media was replenished every 3 days.

On the day of transfection (typically after 5 days in culture), neurons were transfected with HA-βAPP-CFP and LAMP1-mRFP using Lipofectamine 2000. One day following transfection, neurons were surface labeled with Alexa Fluor-647 Zenon-labeled anti-HA antibodies on ice for 30 min. Neurons were then either fixed at 0 min or allowed to internalize at 37 °C for 15 min, after which they were fixed in 4 % paraformaldehyde (PFA) for 15 min. Fixed cells were then imaged using confocal microscopy. Neuronal cultures were stained with NeuN to ensure that 75 % of the cultures were composed of neurons.

### Confocal microscopy

Imaging was performed on a Zeiss LSM-510 META laser scanning microscope using a Zeiss 63X 1.4 numerical aperture oil immersion lens. The optical section thickness was typically 1 micron. mTagBlue was imaged using a 405 nm laser and a BP 390-465 filter. ECFP fluorescence was imaged using 458 nm laser excitation source and a BP 475-525 filter set. EGFP and YFP fluorescence was visualized using a 488 nm excitation laser and a 500-530-nm emission filter set. mRFP fluorescence was imaged using a 543 nm excitation laser and BP 565-615 filter set. Alexa Fluor 647 fluorescence was imaged using 647 nm excitation laser, and a LP 650 filter. The approximate outline of each cell was traced by hand using Adobe Illustrator using the surface labeled HA-fluorescence as a guide; when this was indistinct, the outlines were derived from images which were saturated to show cell edges.

### Antibody cell surface labeling

Anti-HA antibody was labeled with Alexa Fluor 647 using a Zenon mouse labeling kit (Invitrogen) according to the manufacturer’s instructions. For fixed time-course studies, a freshly prepared conjugate was incubated with cells in DMEM on ice for 30 min. The conjugate was removed and the cells were washed twice in pre-warmed HBSS. Following the wash, warm HBSS was added and the cells were incubated at 37 °C for the times indicated prior to fixation with 4 % paraformaldehyde. Cells that were selected for this study showed strong expression of both the APP and compartment marker constructs in addition to normal morphology.

Cell surface crosslinking experiments were adopted from Ehehalt *et al.* [34]. Cells were chilled on ice for 15 min and surface labeled in HBSS containing either a low (5 μg /ml) or high (20 μg /ml) antibody concentration for 1 h on ice. Cells were washed and incubated with Alexa Fluor 647 goat anti-mouse antibody in HBSS at low (1 μg/ml) or high (2.5 μg/ml) concentration for 1 h on ice. Fluorescent dextran, when used, was added at 2 mg/ml. Cells were then either fixed directly or allowed to internalize at 37 °C for 15 min and then fixed with 4 % paraformaldehyde for 15 min.

For live cell imaging, the anti-HA antibody was labeled with Alexa Fluor 647 using a Zenon mouse labeling kit according to the manufacturer’s instructions. Cells were washed twice with warm HBSS. The conjugate was incubated with cells in HBSS for 15 min at room temperature. Conjugate was removed and the cells were washed twice in warm HBSS before being immediately imaged in HBSS at 37 °C on a BC200 microscope stage warmer with a Bionomic BC100 controller (20/20 technologies). Images were taken using a Zeiss 510 META laser scanning confocal microscope at 2 frames/minute in 512 x 512 resolution.

### FlAsH biarsenical fluorescein labeling

Methods were adapted from Taguchi *et al.* [47]. Labeling media consisted of 1.4 uM FlAsH reagent (sold under the trade name “Lumio” by Invitrogen), 1 mM 1,2-ethanedithiol (EDT) (Sigma) and 20 mM DL-Dithiothreitol (DTT) (Sigma) incubated together in the dark at room temperature in HBSS for 10 min. SN56 cells were washed and labeled on ice for 3 min. After labeling, cells were washed 2x with room temperature 250 uM EDT in HBSS. The final wash was done with warm HBSS and the cells were allowed to internalize at 37 °C for 15 min. Following internalization, cells were fixed for 15 min in 4 % PFA and imaged on an LSM510 confocal microscope (Carl Zeiss). For FlAsH and anti-HA combination labeling, following treatment with FlAsH labeling media, cells were treated with 1:100 Alexa Fluor-647 Zenon-tagged anti-HA antibodies for 30 min following FlAsH labeling. After labeling with Zenon, cells were washed 2x in warm HBSS and allowed to internalize for 15 min before being fixed.

### Data quantification and analysis

Co-localization analysis was performed on confocal optical sections using Imaris 7.0.0 with Imaris Co-localization module (Bitplane) running on an Apple Mac Pro to examine the co-localization of the brightest 2 % of pixels in each channel of interest, often between HA-tagged APP and Lamp1-mRFP or Rab5-RFP. This allows us to set a threshold for co-localization in an unbiased manner using the intrinsic properties of the image, eliminating confounding problems caused by variations in cell-to-cell expression and image brightness/exposure thus allowing direct comparison between experiments. This is discussed more fully in our previous work Lorenzen *et al.* [25] as well as in studies by Hutcheon *et al.* [36]; Hutcheon *et al.* [84]. Graphing and statistical analysis was performed using Prism GraphPad 5.01 using one-way ANOVA with Tukey post-test or t-tests as indicated.

### siRNA knockdowns

SN56 cells were split as described in cell culturing subsection. Stealth siRNAs (Invitrogen) were purchased for Arf6 (AUAAUGCGGUGCAGCUCCUGGCGGG) (adapted from Bach et al. 2010 for use in mouse), Arf1 (GGGAAUAUCUUUGCAAACCUCUUCA) and Rac1 (GCCUGCUCAUCAGUUACACGACCAA). Cells were transfected with increasing amounts of siRNA and western blotted to demonstrate knockdown. Cell lysates were collected 3 days after transfection and assayed by western blotting with a 1:1000 concentration of Anti-Rac1, Anti-Arf1 or Anti-Arf6 antibodies.

For trafficking studies, cells were transfected with HA-βAPP-CFP, LAMP1-mRFP and either 300 nM of Arf6 siRNA or 500 nM of Arf1 siRNA or a combination of both. Rac1 siRNA was used at 400 nM. Cells were differentiated 1 day following transfection and were then surface labeled with Alexa Fluor-647 Zenon-labeled anti-HA antibodies as described above and allowed to internalize at 37 °C for 15 min.

### Protein extraction and western blotting

SN56 cells (1.5 x 10^6^) were plated on 60 mm dishes (Nunclon) and transfected with the appropriate DNA constructs/siRNA transcripts. Following 3 days of incubation, cells were washed in cold PBS and lysed with NP40 lysis buffer (20 mM Tris pH 8.0, 137 mM NaCl, 10 % glycerol, 1 % IGEPAL/NP40) for 5 min at 4 °C. Cells were then scraped and centrifuged at 14,000 rpm for 15 min at 4 °C to remove insoluble material. Total cell lysates were separated by SDS-PAGE and transferred onto PVDF (polyvinylidene fluoride) membrane.

PVDF membrane was then probed with Arf1 (1:1000), Arf6 (1:1000) or α-tubulin (1:10,000) antibodies (Sigma), developed using ECL and exposed to film. Quantification of western blot exposures was accomplished using ImageJ.

### Electron microscopy

SN56 cells were plated on 35 mm confocal dishes and transfected with HA-βAPP-CFP and LAMP1-mRFP. Cells were first labeled on ice with 1:20 concentration of anti-HA antibody for 30 min. This was followed by labeling on ice with 1:20 concentration of 10 nm Alexa Fluor 488 colloidal gold conjugate (Invitrogen) for 30 min. Cells were then incubated for 15 min in warm HBSS and then fixed in 2 % glutaraldehyde in 0.1 M Na phosphate buffer pH 7.2 for 15 min and then with 2 % osmium tetroxide, in 0.1 M Na phosphate Buffer pH 7.2 in the cold for 30 min. Cells were dehydrated and introduced into Polybed 812 through an ethanol series, and then allowed to harden overnight at 60 °C in culture dishes. Precast resin cylinders are glued over areas populated with cells with plastic resin and allowed to harden at 60 °C overnight. The cover glass was removed from the plate by floating on concentrated hydrofluoric acid for 15 min followed by washes in 2 N sodium hydroxide and distilled water. Blocks were separated with a fine jeweler’s saw. Ultrathin sections (80 nm) were cut using a Reichert Ultracut S with a Diatome diamond knife and sections were collected on 200 mesh copper grids and left unstained, or stained with 2 % alcoholic Uranyl acetate followed with staining in Reynolds Lead citrate. Sections were viewed at 60 K volts with a Philips 410 electron microscope equipped with an ATM digital camera system.

### Immunohistochemistry

Formalin-fixed Post Mortem human tissue was obtained from the London Health Science Centre University Hospital Pathology Department after review from the hospital Tissue Use Committee (#TA 217) and the University of Western Experiment Research Ethics Board (UWO Ethics Study #: 16256E), after formal neuropathological diagnosis was established. Tissue blocks were taken through the hippocampus, embedded in paraffin and 6-μm sections were cut. Slides were deparaffinized in xylenes and rehydrated in graded ethanols. Antigen retrieval was performed by immersing slides for 5 min in boiling Sodium Citrate Buffer (10 mM trisodium citrate with 0.05 % Tween 20 pH 6.0). Endogenous peroxidase activity was quenched with 3 % H2O2 and the nonspecific binding sites blocked (5 % BSA, 0.3 % Triton X-100 in PBS). Primary antibody incubation was performed for 20h at 4 °C, followed by incubation with the appropriate biotinylated Anti-rabbit secondary antibody for 60 min at RT and detection was performed (Vectastain Elite ABC Kit, Vector Laboratories, Burlingame, CA) using the manufacturer’s directions, followed by counterstaining with modified Harris Haematoxylin. Images were taken on an Olympus BX 45 light microscope an Aperio ScanScope Slide Scanner.

### Aβ42 and Aβ40 ELISA

SN56 cells were plated into 12-well plates and then transfected with either HA-βAPP-CFP or βAPP-Swedish-CFP and one of Arf1-DN, Arf6-DN or Arf6-CA mutant constructs. After 3 days in culture, 500 μl of media was collected and assayed using an ultrasensitive Aβ42 or Aβ40 ELISA kit (Invitrogen) according to the manufacturer’s instructions. Data was plotted and analyzed using Graphpad Prism 5.0 software.

## Additional files

Additional file 1: Figure S1. Demonstration of co-localization channel in Imaris and that lysosomal transport of APP occurs in neurons and is not influenced by the APP construct or the presence of epitope or fluorescent tags. A) Mouse cortical cultures were transfected with HA-βAPP-CFP and Lamp1-mRFP (red), surface labeled with Alexa Fluor 647 anti-HA antibodies (green) on ice, and then fixed or incubated at 37 °C for 15 min. After 15 min at 37 °C. Imaris software was used to set thresholds are set to identify the brightest 2 % of pixels and these are used to generate a colocalization channel (white) demonstrating APP in a LAMP1 labeled compartment. After 15 min, surface labeled APP is present in a LAMP1 positive compartment. Approximate outline of the cells is superimposed for clarity. B) SN56 cells were transfected with the APP construct indicated, surface labeled with Alexa Fluor 647 labeled antibodies (green) which was allowed to internalize for 15 min. Lysosomes were identified using either transfected LAMP1-mRFP or by immunostaining LAMP2 (red). Colocalized pixels were identified using Imaris Software and overlayed in white. The HA-βAPP constructs internalized to lysosomes as identified by transfected LAMP1-mRFP and by LAMP2 staining. Full length APP-695 (with no C- or N- terminal tag) is labeled by the anti-amyloid antibody 6E10 and transported to LAMP2 compartments. Approximate outline of the cells is superimposed for clarity. Scale bar = 5 microns. Additional file 2: Video S1A. Live Cell Video Demonstrating Rapid Internalization of APP to the lysosome (From Fig. 1). SN56 cells were transiently transfected with HA-βAPP-CFP (not shown) and LAMP1-RFP (red) and surface labeled on ice for 30 min with Alexa Fluor 647 labeled anti-HA antibodies. Cells were then transferred to a heated microscope stage and image approximately every 30 s. **Video S1B.** Same as Video S1A with brightfield DIC channel overlayed. Additional file 3: Figure S2. APP N-Terminal HA-epitope tag and C-terminal Fluorescent protein tag are co-transported. SN56 cells were transfected with HA-βAPP-CFP (cyan) and LAMP1-mRFP (red), surface labeled with Alexa Fluor 647 anti-HA antibodies (green) on ice for 30 min, and then transferred to the microscope stage and imaged. A) A whole SN56 cell with sub-region of interest indicated with a box. Scale bar = 5 microns. B) Demonstrates the image time course from inset box in A. Images show the 3 color image (top line), merged HA and APP-CFP (green and cyan) (middle row), and merged LAMP1 and HA (red and green) (bottom row) Scale bar = 1 micron. Arrowheads indicate APP-CFP colocalized with anti-HA-antibody, while arrows indicate all where anti-HA, APP-CFP and LAMP1-mRFP are co-localized. Elapsed time (min:sec) is shown. Additional file 4: Video S2. 3D Stack of a Neuron demonstrating that cell surface APP is present in lysosomes within 15 min. Cultured mouse neurons were transiently transfected with HA-βAPP-CFP (not shown) and LAMP1-RFP (red) and surface labeled on ice for 30 min with Alexa Fluor 647 labeled anti-HA antibodies (green). Cells were allowed to internalize at 37° for 15 min, fixed, and 3D stacks of images were taken by confocal microscopy. Images were reconstructed using Imaris 7.0 software. The first sequence shows the nucleus in blue and lysosomes in red. The next sequence overlays internalized APP in green. Finally, a white colocalization channel is superimposed. Additional file 5: Video S3. 3D Stack of a Neuron demonstrating that Rapid Lysosomal transport of APP occurs even in large neurons, in processes and cell bodies. Cultured mouse neurons were transiently transfected with HA-βAPP-CFP (not shown) and LAMP1-RFP (red) and surface labeled on ice for 30 min with Alexa Fluor 647 labeled anti-HA antibodies. Cells were allowed to internalize at 37° for 15 min, fixed, and 3D stacks of images were taken by confocal microscopy. Images were reconstructed using Imaris 7.0 software. The first sequence shows the green internalized APP, followed by green and red channels together. Finally, the white co-localization channel generated by Imaris software is superimposed. Additional file 6: Figure S3. Antibody binding increased APP internalization. A) SN56 cells were transiently transfected and incubated on ice with low (5 μg /ml) or high (20 μg /ml) concentrations of anti-HA antibodies. These antibodies were further cross-linked on ice with a fluorescently labeled secondary antibody, allowed to internalize for 15 min, and then fixed and imaged. Co-localized pixels are overlayed in white. Scale bar = 5 microns. B) Colocalization of APP was quantitated. After 15 min at 37°, 12.5 ± 0.53 % of the surface labeled APP was internalized at the low antibody concentration, and this doubled to 22.1 ± 0.41 % at the higher antibody concentration (*P* < 0.05). Additional file 7: Video S4. Live Cell Video Demonstrating Rapid Internalization of APP to the lysosome is blocked by transfection of an Arf6-Dominant negative construct. SN56 cells were transiently transfected with HA-βAPP-CFP (not shown), LAMP1-RFP (red) and Arf6-T27N-DN (not shown) and surface labeled on ice for 30 min with Alexa Fluor 647 labeled anti-HA antibodies. Cells were then transferred to a 37 °C heated microscope stage and imaged approximately every 30 s. Co-localized pixels are overlayed in white.

## Abbreviations

LAMP1: Lysosomal Associated Membrane Protein 1
LAMP2: Lysosomal Associated Membrane Protein 2
APP: Amyloid Precursor Protein
Aβ: beta-amyloid peptide
HA tag: hemagglutinin epitope for antibody binding
CFP: Cyan Fluorescent Protein
mRFP: monomeric red fluorescent protein
YFP: Yellow Fluorescent Protein
