# Supplementary figures and images for: Arf6 controls beta-amyloid production by regulating macropinocytosis of the Amyloid Precursor Protein to lysosomes
Source: Mol Brain. 2015 Jul 14;8:41. doi: 10.1186/s13041-015-0129-7 (PMC4501290; doi:10.1186/s13041-015-0129-7)

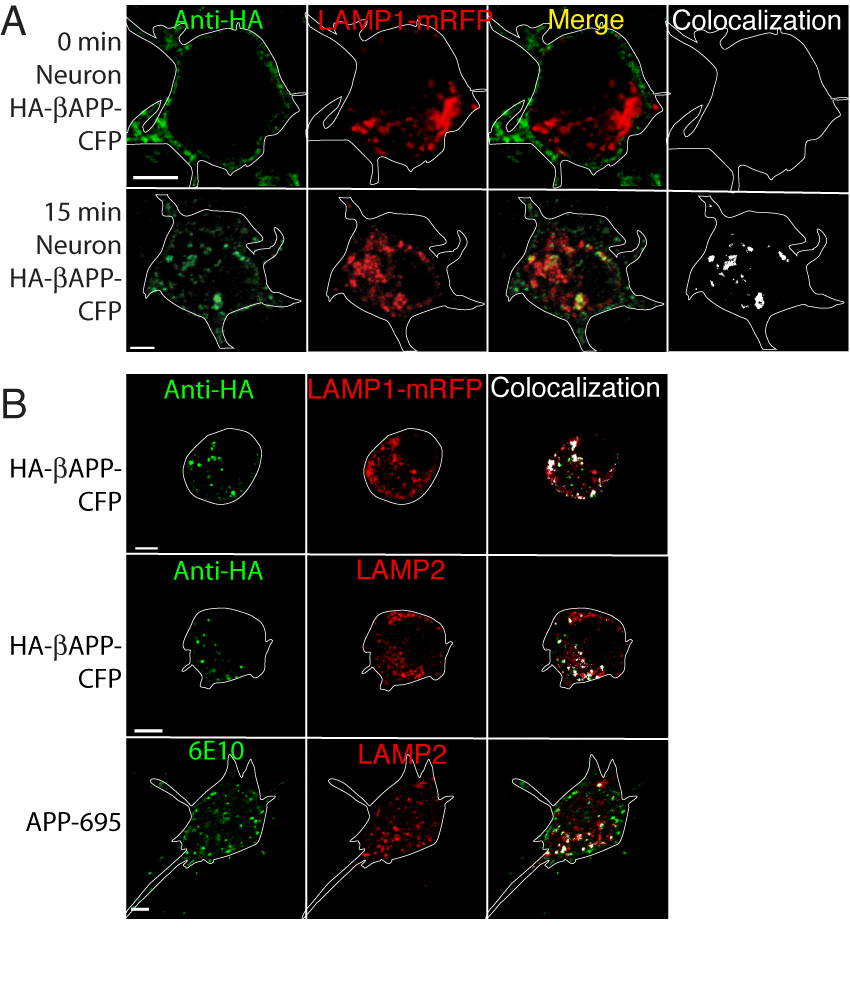

Supplement: Additional file 1: Figure S1. — Demonstration of co-localization channel in Imaris and that lysosomal transport of APP occurs in neurons and is not influenced by the APP construct or the presence of epitope or fluorescent tags. A) Mouse cortical cultures were transfected with HA-βAPP-CFP and Lamp1-mRFP (red), surface labeled with Alexa Fluor 647 anti-HA antibodies (green) on ice, and then fixed or incubated at 37 °C for 15 min. After 15 min at 37 °C. Imaris software was used to set thresholds are set to identify the brightest 2 % of pixels and these are used to generate a colocalization channel (white) demonstrating APP in a LAMP1 labeled compartment. After 15 min, surface labeled APP is present in a LAMP1 positive compartment. Approximate outline of the cells is superimposed for clarity. B) SN56 cells were transfected with the APP construct indicated, surface labeled with Alexa Fluor 647 labeled antibodies (green) which was allowed to internalize for 15 min. Lysosomes were identified using either transfected LAMP1-mRFP or by immunostaining LAMP2 (red). Colocalized pixels were identified using Imaris Software and overlayed in white. The HA-βAPP constructs internalized to lysosomes as identified by transfected LAMP1-mRFP and by LAMP2 staining. Full length APP-695 (with no C- or N- terminal tag) is labeled by the anti-amyloid antibody 6E10 and transported to LAMP2 compartments. Approximate outline of the cells is superimposed for clarity. Scale bar = 5 microns. [file 13041_2015_129_MOESM1_ESM.jpeg]

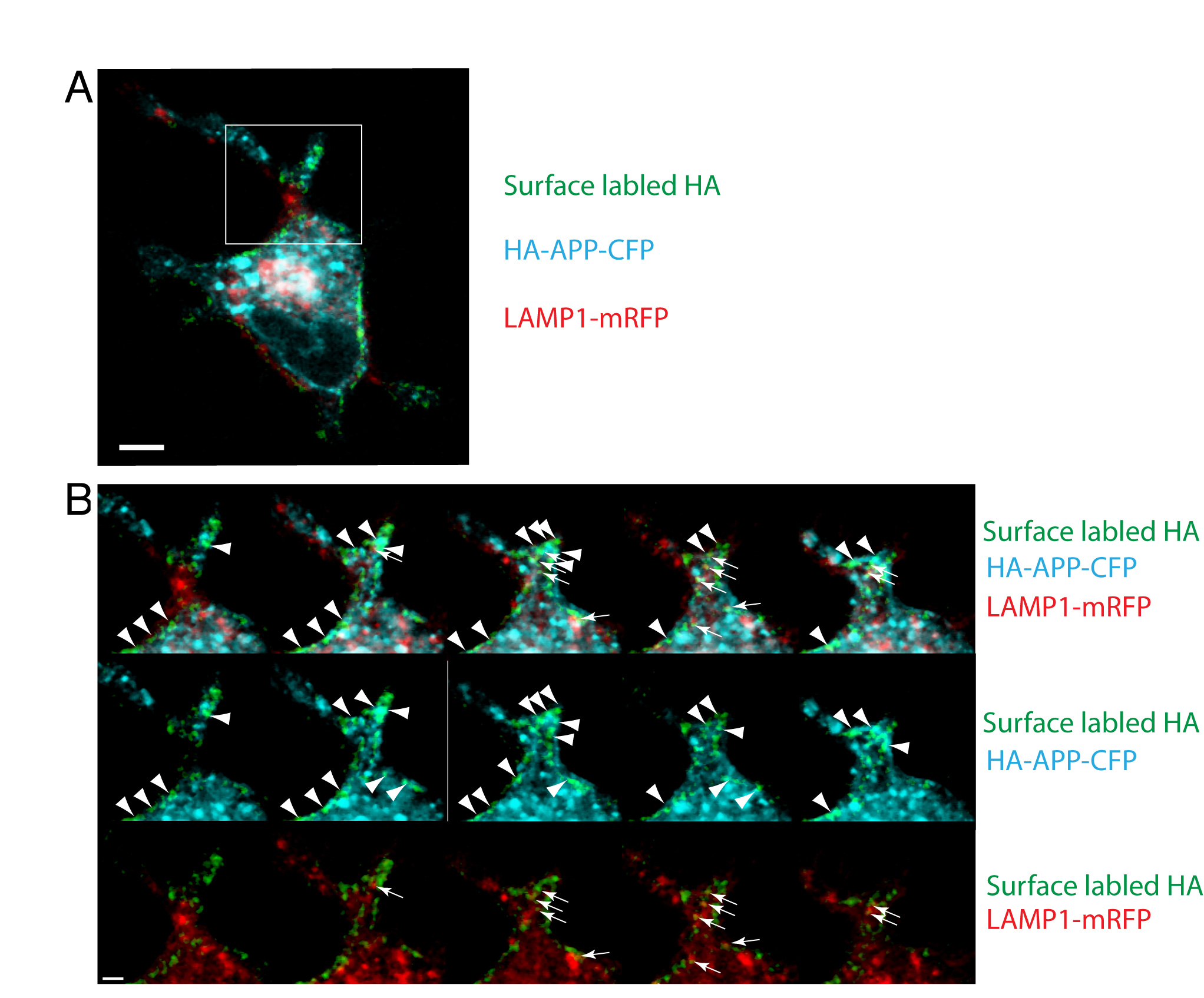

Supplement: Additional file 3: Figure S2. — APP N-Terminal HA-epitope tag and C-terminal Fluorescent protein tag are co-transported. SN56 cells were transfected with HA-βAPP-CFP (cyan) and LAMP1-mRFP (red), surface labeled with Alexa Fluor 647 anti-HA antibodies (green) on ice for 30 min, and then transferred to the microscope stage and imaged. A) A whole SN56 cell with sub-region of interest indicated with a box. Scale bar = 5 microns. B) Demonstrates the image time course from inset box in A. Images show the 3 color image (top line), merged HA and APP-CFP (green and cyan) (middle row), and merged LAMP1 and HA (red and green) (bottom row) Scale bar = 1 micron. Arrowheads indicate APP-CFP colocalized with anti-HA-antibody, while arrows indicate all where anti-HA, APP-CFP and LAMP1-mRFP are co-localized. Elapsed time (min:sec) is shown. [file 13041_2015_129_MOESM3_ESM.jpeg]

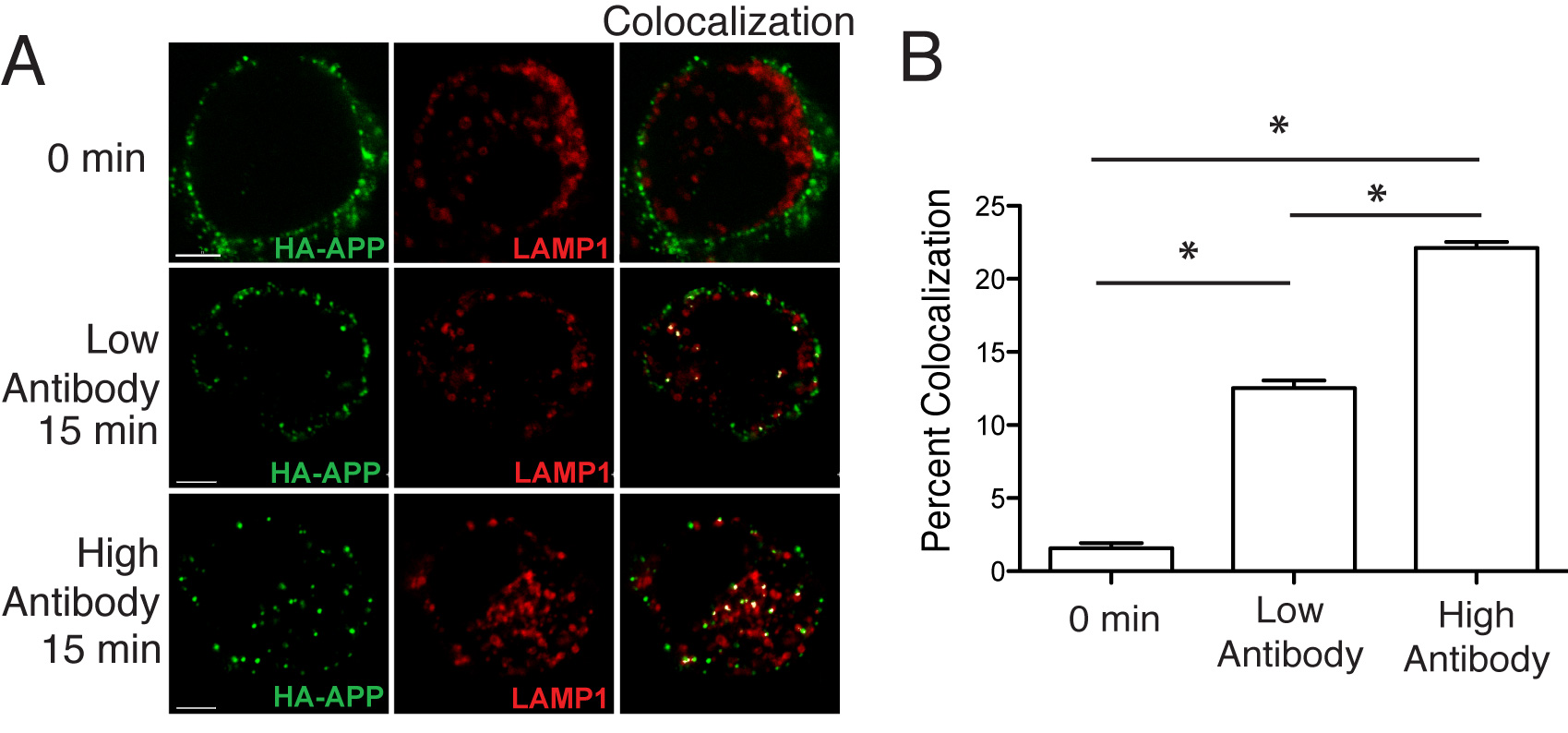

Supplement: Additional file 6: Figure S3. — Antibody binding increased APP internalization. A) SN56 cells were transiently transfected and incubated on ice with low (5 μg /ml) or high (20 μg /ml) concentrations of anti-HA antibodies. These antibodies were further cross-linked on ice with a fluorescently labeled secondary antibody, allowed to internalize for 15 min, and then fixed and imaged. Co-localized pixels are overlayed in white. Scale bar = 5 microns. B) Colocalization of APP was quantitated. After 15 min at 37°, 12.5 ± 0.53 % of the surface labeled APP was internalized at the low antibody concentration, and this doubled to 22.1 ± 0.41 % at the higher antibody concentration (P < 0.05). [file 13041_2015_129_MOESM6_ESM.jpeg]
